# Anti-inflammatory effects of H_2_S during acute bacterial infection: a review

**DOI:** 10.1186/s12967-017-1206-8

**Published:** 2017-05-10

**Authors:** Francesca Benedetti, Sabrina Curreli, Selvi Krishnan, Sergio Davinelli, Fiorenza Cocchi, Giovanni Scapagnini, Robert C. Gallo, Davide Zella

**Affiliations:** 1 Institute of Human Virology, University of Maryland School of Medicine, Baltimore, MD 21201 USA; 2 Department of Medicine and Health Sciences, University of Molise, 86100 Campobasso, Italy

**Keywords:** Mycoplasma, Hydrogen sulfide, NaHS, GYY4137, NF-kB, Nrf2, ROS, Inflammation, U937

## Abstract

Hydrogen sulfide (H_2_S), previously only considered a toxic environmental air pollutant, is now increasingly recognized as an important signaling molecule able to modulate several cellular pathways in many human tissues. As demonstrated in recent studies, H_2_S is produced endogenously in response to different cellular stimuli and plays different roles in controlling a number of physiological responses. The precise role of H_2_S in inflammation is still largely unknown. In particular, the role of H_2_S in the regulation of the inflammatory response in acute and chronic infections is being actively investigated because of its potential therapeutic use. To study the effect of H_2_S as an anti-inflammatory mediator during bacterial infections, we developed an ex vivo model of primary cells and cell lines infected with Mycoplasma. Our data demonstrate a dichotomic effect of H_2_S on the NF-kB and Nrf-2 molecular pathways, which were inhibited and stimulated, respectively.

## Background

There is a growing interest in “medical gasses” for their antibacterial and anti-inflammatory properties. In this review, we focus on hydrogen sulfide (H_2_S), an endogenous gaseous mediator that has gained increasing recognition as an important player in modulating acute and chronic inflammatory diseases. Therefore, understanding the H_2_S-mediated mechanism of action during the inflammatory response to infection/s is essential for developing H_2_S-releasing compounds as candidate drugs [1].

Among the numerous effects attributable to H_2_S, many studies, both in vivo and in vitro, have demonstrated its protective anti-inflammatory role in lung pathologies such as asthma and COPD [2]. In addition, it has been observed a cardio-protective effect [3], together with pro-angiogenic [4] and vasorelaxing effects mediated by KATP channel opening [5, 6]. Other studies also demonstrated the inhibitory effect of H_2_S on platelet aggregation [7] and its antiapoptotic [8] and cytoprotective action [9]. Recently, endogenous H_2_S production and exogenous H_2_S administration were both demonstrated to play an important role in modulating viral-induced chemokine secretion and viral replication [10, 11].

However, it’s not well known whether H_2_S impacts the outcome of bacterial infection.

In this review we focus mainly on the effects of H_2_S during Mycoplasma infection. Mycoplasma was used as a bacterial model of acute infection, since it has been associated with the onset and the progression of several human pathologies [12–15]. We also discuss the potential use of H_2_S-releasing compounds as candidate drugs able to relieve the inflammatory response caused by Mycoplasma.

## Mycoplasmas

Mycoplasmas (class Mollicutes, mollis = soft and cutis = skin, in Latin) are the smallest and simplest organized prokaryotic organisms, ranging from 0.1 to 0.3 μm in diameter and up to 98 μm in length, capable to self-reproduction [16]. In contrast to the rest of the bacteria, Mycoplasma do not have a cell wall, but have a simple plasma membrane composed of sterols. Mycoplasmas display a dominant spherical shape, modulated by the presence of a cytoskeleton, that contributes also to cell division (the reproduction occurs by binary fission) and to their motility [17].

Mycoplasmas have a circular double-stranded genome, with a low guanine-cytosine (G + C) content, that is very variable among strains of the same Mycoplasma species [18]. The variability is due to repetitive elements, consisting of segments of protein genes, different in size and number, or insertion sequence elements (IS). The size is limited and ranges from less than 600–2200 kb. Because of their small genome size, Mycoplasma have restricted metabolic capabilities and their replication and survival depend on factors synthesized by the host in vivo or taken up by the growth medium in vitro [19]. Mycoplasma species are parasites that can be found everywhere in nature. In the human body they usually exhibit organ and tissue specificity, with preferential localization in the mucous surface of the respiratory and urogenital tracts, in the eyes, in the alimentary canal, in the mammary glands and in the joints [17]. Mycoplasma are considered membrane surface parasites, however some species can enter the cells and become intracellular residents [20, 21].

## Mycoplasmas and human diseases

Although most Mycoplasmas belong to the normal human bacterial flora and thus considered commensal inhabitants, a few species are pathogens, with a significant negative impact on the cellular metabolism and physiology. They can be both associated with infectious diseases and post-infection pathologies, and frequently persist as chronic, asymptomatic infections both in humans and animals [22]. Mycoplasmas can cause a wide variety of diseases, including acute respiratory illness [15], genitourinary tract infections [23], joint infections [13, 14] and neurologic disorders [24].

Seven species of Mycoplasma are related to human pathologies: *Mycoplasma pneumoniae, Mycoplasma urealyticum, Mycoplasma genitalium, Mycoplasma hominis, Mycoplasma fermentans, Mycoplasma penetrans and Mycoplasma pirum* [25]. The mechanisms responsible for their potential pathogenic role are the subject of a number of current investigative efforts. Deregulated cellular activation leading to production of pro-inflammatory cytokines plays an important role in bacterial-induced pathologies. Though they lack lipopolysaccharides (LPS), all Mycoplasma species activate monocytes-macrophages, lymphocytes and fibroblasts through membrane-bound lipoproteins present in their bacterial membranes which act as agonists of the heterodimeric Toll-like receptors 2 and 6 (TLR2 and TLR6) [26]. Consequently, by regulating specific transcription factors, they promote the expression of pro-inflammatory cytokines, thus affecting several important cellular functions [17, 27–29].

Mycoplasma is part of the human microbiome, and epidemiological studies and recent genomic sequence analysis clearly indicate the involvement of human microbiome in early stages of cellular transformation and cancer progression [30]. The presence of Mycoplasma may facilitate tumorigenesis by promoting cellular transformation [31, 32], as shown by many studies in vitro in bronchial epithelial cells [33], in hepatocytes [34], in oral tissues [35], in human prostate cells [36, 37] and cervical cells [38]. Although the molecular basis of Mycoplasma’s oncogenic potential are still under investigation, a few studies have demonstrated increased frequency of chromosomal instability and malignant transformation in long-term Mycoplasma infected cell cultures. The Mycoplasmas involved (*Mycoplasma fermentans, Mycoplasma penetrans and Mycoplasma hyorhinis*) not only caused accumulation of chromosomal abnormalities, but also phenotypic changes of the transformed cells [39–41]. In other studies, long-term infection of mouse embryo fibroblasts with *Mycoplasma fermentans* or *Mycoplasma penetrans* demonstrated spontaneous cellular transformation and overexpression of the H-Ras and c-myc proto-oncogenes [42]. Consistent with its proposed role in cellular transformation, Mycoplasma infection reduced activation of p53 and induced constitutive activation of NF-κB [43].

Another factor involved in DNA stability is DNA methylation. DNA methylation is an essential element in transcriptional regulation and is one of the major epigenetic mechanisms leading to DNA remodeling. Many stress-inducing factors and/or DNA-damaging agents can interfere with the effectiveness of the DNA-methyltransferases (DNA-MTases) to modify the DNA by converting cytosine to 5-methylcytosine at CpG dinucleotides [44]. In this regard, it was demonstrated that when Mycoplasma hyorhinis’s CG- and GATC-specific MTase is expressed in human cell lines, it translocates to the nucleus, contributing to the methylation process. This unprogrammed change in the human genome landscape has been associated with the stimulation of pro-oncogenic pathways [45]. However, so far no carcinogenic roles for any Mycoplasma have been demonstrated in vivo, where Mycoplasmas have been isolated and only associated to cancer by analyzing different specimens (infectious tissues, neoplastic tissues and body fluids) from patients, without any demonstration of a causative effect. In particular, Mycoplasmas have been found in precancerous lesions as well as in malignant tissues from patients with stomach, colon, ovarian and lung cancers [46].

## NF-κB and Nrf2: two molecular pathways involved in inflammation

The inflammatory response is characterized by the coordinated activation of various signaling pathways that regulate expression of both pro- and anti-inflammatory mediators in resident tissue cells and leukocytes recruited from the blood. Arguably, the most important of these pathways involves the nuclear factor kappa B (NF-κB) family of transcription factors, acting as master regulators of immune and inflammatory processes in response to both injury and infection.

Nuclear factor kappa B is considered a “rapid-acting” primary transcription factor: it is always present in the cells in an inactive state and does not require new protein synthesis in order to become activated. For this reason it is among the first responders to cellular and exogenous stimuli such as stress, cytokines, free radicals, ultraviolet irradiation and ionizing radiation, oxidized LDL, bacterial or viral antigens and reactive oxygen species (ROS) [47, 48]. The mammalian NF-κB family includes five proteins: NF-κB1 (p50), NF-κB2 (p52), RelA (p65), RelB and c-Rel. All these proteins share a Rel homology domain in their N-terminus. RelA, RelB, and c-Rel have a transactivation domain in their C-termini. In contrast, the NF-κB1 and NF-κB2 proteins are synthesized as large precursors (p105 and p100), and they need to be processed by the ubiquitin/proteasome pathway to generate the mature NF-κB subunits, p50 and p52, respectively. Two NF-κB signaling pathways exist in the cells: the classical (canonical) pathway and the alternative (non-canonical) pathway [47]. In unstimulated cells, the NF-κB dimers are sequestered in the cytoplasm by a family of inhibitors, called IκBs (Inhibitor of κB). These proteins contain multiple copies of a sequence called ankyrin repeats and mask the nuclear localization signals (NLS) of NF-κB proteins to keep them sequestered in an inactive state in the cytoplasm. Its activation is initiated by the signal-induced degradation of IκB proteins, via activation of a kinase called IKK (IκB kinase). Upon degradation of IκB, the NF-κB complex translocates to the nucleus where it binds to specific DNA motifs, eventually resulting in the expression of specific genes involved in several physiological responses, including inflammatory response, cellular development, maturation, survival and proliferation [49]. Furthermore, NF-κB plays a key role both in regulating the immune response to infection and in the processes of synaptic plasticity and memory [50]. By turning on the expression of its own repressor IκBα, NF-κB activity is self-regulated through an auto feedback loop [51].

As discussed previously, NF-κB is responsible for the transcription of many genes involved in inflammation. It is thus not surprising that it is found to be chronically active in many inflammatory diseases, such as inflammatory bowel disease [52], arthritis [53], and asthma [54].

Together with the inflammatory response, NF-κB activation is involved in the control of apoptosis. In fact, upon nuclear translocation, NF-κB induces the transcription of anti-apoptotic proteins that lead to increased cellular proliferation (cyclins and CDKs), angiogenesis (VEGF, IL-6, MCP-1 and MMPs), and invasion and metastasis (VCAM-1, ICAM-1, MMPs or proteins involved in the maintenance of the epithelial mesenchymal transition) [55, 56]. Indeed, constitutive activation of NF-κB is observed in a wide variety of cancers, such as lymphoma, liver cancer, lung cancer and breast cancer [57]. Furthermore, by positively affecting the expression of the major inflammatory factors, such as TNFα, IL-6, IL-1 and IL-8, constitutive activation of NF-κB contributes to creating a microenvironment favorable for tumor cells: chronic inflammation is in fact implicated in all stages of cancer development and progression [58, 59]. In general, chronic inflammation, as defined by elevated levels of both local and systemic cytokines and other pro-inflammatory factors, is a hallmark of aging in virtually all higher animals including humans and is recognized as a major risk factor for developing age-associated diseases.

Nuclear factor-E2-related factor 2 (Nrf2) is another nuclear factor critically involved in inflammation, belonging to a family of cap’n’collar proteins that regulates the endogenous antioxidant defense. It promotes the transcription of a set of detoxifying genes (ARE, antioxidant response elements) codifying for proteins (such as enzymes, drug transporters, antiapoptotic proteins and proteasomes) involved in the regulation of physiological and pathophysiological cellular events following exposure to oxidant and xenobiotics agents [60]. Therefore Nrf2 activation results in a protective activity against cellular damage(s) potentially leading to a number of human pathologies, such as cancer, neurodegenerative diseases, cardiovascular diseases, acute lung injury, chronic obstructive pulmonary diseases, autoimmune diseases, infection and inflammation [61].

Depending on the context of cellular stimulation, activation and/or differentiation, two forms of Nrf2 are observed: inactive or active. In its inactive form, Nrf2 is retained in the cytoplasm associated with Keap1, a cytoskeletal protein that interacts directly with actin [62], is ubiquitinated, and targeted for degradation by the 26S proteasome. Also Keap1 binds to Cul3 to promote directly the degradation of Nrf2 [63]. In response to cellular insults, like in the presence of ROS (reactive oxygen species), the redox-stress sensitive cysteine residues in Keap1 are modified, Nrf2 is released from its repressor and translocates to the nucleus, where it forms heterodimers with bZIP proteins such as small musculoaponeurotic fibrosarcoma (Maf) proteins [64]. This heterodimer recognizes and binds to a specific sequence element called “antioxidant electrophile responsive element” (ARE) [63]. Other proteins, such as c-Jun [65] and ATF4 [66], are able to interact with Nrf2 as molecular partners to promote its transcriptional activity. As a result, the binding of the heterodimer to the ARE elements promotes the transcription of many genes which codify for proteins with different key cellular defensive functions, thus enhancing the removal of cytotoxic electrophiles or ROS [67]. These proteins include NADPH quinone oxidoreductase (NQO1), glutathione S-transferase (GST), heme oxygenase-1 (HO-1), superoxide dismutase (SOD), glutathione peroxidase (GPx), catalase (CAT), GSH reductase (GR), glutamate cysteine ligase (GCL), peroxiredoxin I (PrdxI) and and γ-glutamycysteine synthase.

Furthermore, besides its protective role against oxidative and electrophilic stresses, recent studies have demonstrated that Nrf2 responds to pro-inflammatory stimuli by inhibiting the production/expression of pro-inflammatory mediators including cytokines, chemokines, cell adhesion molecules, matrix metalloproteinases, cyclooxygenase-2 and inducible nitric oxide synthase [68]. In the case of Mycoplasma infection, it has been shown that Nrf2 activation plays a relevant role in the modulation of the downstream inflammatory response [69]. Many other researches have demonstrated, in different cell types and tissue contexts, that Nrf2 induction can inhibit the NF-κB pathway and thus indirectly modulate inflammatory cytokines and chemokines signaling [70]. This inhibition of NF-κB likely occurs through ARE-driven reduction in oxidative stress, which has been shown to activate the NF-κB signaling pathway [71]. Further supporting a role for Nrf2, several studies have demonstrated increased NF-κB activation and dysregulation of cytokines and chemokines in Nrf2^−/−^ mice after inflammatory various insults [72].

Based on its multiple anti-inflammatory functions, Nrf2 is currently used as a pharmacological and nutritional target to prevent and treat chronic diseases, such as multiple sclerosis, chronic kidney diseases and cardiovascular diseases [73, 74].

## Hydrogen sulfide (H_2_S)

The gasotransmitters family includes nitric oxide (NO), carbon monoxide (CO) and hydrogen sulfide (H_2_S). In particular, H_2_S, commonly found in nature, especially dissolved in the hydrothermal water [75], is increasingly being recognized as an important signaling molecule in the regulation of the cellular metabolism, the immunological and inflammatory responses, and several important transcription factors [76].

H_2_S is a ubiquitous gas produced endogenously in the human body in all tissues, the highest production being in the brain, in the cardiovascular system, in the liver and in the kidney [77]. H_2_S is produced during cysteine metabolism mediated by various non-enzymatic and enzymatic steps involving key enzymes CBS (cystathionine-β-synthase) and CSE (cystathionine-γ-lyase) [78]. H_2_S being a gasotransmitter, it does not require specific transporters or receptors. It travels rapidly through the cell membranes, exerting multiple biological effects on various biological targets.

As a gaseous signaling molecule, H_2_S freely diffuses across cell membranes in a receptor-independent manner and activate various cellular targets, exerting many different biological effects (from cytotoxic effects to cytoprotective actions). This distinct ability makes H_2_S an attractive pharmacological agent for the treatment of different disease conditions. Many studies showed H_2_S involvement in several physiological and pathological contexts, including oxidative stress regulation via scavenging reactive oxygen species, inflammation, vasodilation, and neuronal survival [79]. In this regard, data obtained using many different cell types have shown its effects on the cell viability, proliferation, activation, cytokines secretion and cell adhesion [1]. H_2_S has widely been demonstrated to have cardio-protective [3] and pro-angiogenic effects [4, 80] both in in vivo and in vitro, and also to have inhibitory effects on platelet aggregation [7], antiapoptotic activity [8] and cytoprotective effects [9].

Moreover, H_2_S acts as vasorelaxant molecule through a mechanism involving the opening of smooth muscle KATP channels with the consequent increased ionic flux, resulting in membrane hyperpolarization [6]. This activity also explains the cardio-protective properties of H_2_S [81].

A protective effect on neuronal cells and also an activation of primary afferent neurons by soluble H_2_S have been reported, making H_2_S worthy of consideration as a new neurotransmitter [82].

The potential modulation of cancer incidence and progression by H_2_S-releasing drugs has been shown in different types of tumors. Nevertheless, there are some other studies that suggest a promotion of tumor growth under some circumstances, presumably because of its effects on angiogenesis [83].

Recent studies focused on lifespan elongation, reported a regulatory role of H_2_S (50 ppm) in *C. elegans* ageing, involving both direct and indirect mechanisms [84]. Some key regulatory molecules, such as Sirtuins and Klotho, contribute to the direct effects of H_2_S, whereas the anti-oxidative and anti-inflammatory nature of H_2_S might protects the ageing of cells and tissues indirectly [85].

The precise role of H_2_S in inflammation is still far from clear. In fact, many studies are trying to understand its precise molecular mechanism(s) of action and its biological relevance, since it appears to have pro- or anti- inflammatory effects under different conditions. This dichotomy may be dependent on dose–response relationship and suggests that H_2_S affects different cellular pathways as well as different cellular targets [86]. For example, some data showed that H_2_S donors have the ability of suppressing leukocyte adherence to the vascular endothelium, with the consequent reduction of the infiltration to the sites of inflammation [87], while others showed their ability to promote the survival of cultured granulocytes by the inhibition of caspase-3 cleavage and p38 phosphorylation [88]. In our previous study we tested the anti-inflammatory effects of H_2_S in an in vitro model of macrophages infected with Mycoplasma, a pro-inflammatory micro-organism capable of triggering the rapid recruitment of a large number of macrophages especially in the lung and airways [89]. Our data showed that exogenous H_2_S is able to inhibit the activation and the nuclear translocation of NF-κB, reducing the transcription of pro-inflammatory genes and the production of several pro-inflammatory cytokines (including MCP-1 gene).

## New findings

Based on the results obtained while studying the effects of H_2_S on the NF-κB pathway, we wanted to further elucidate the role of Nrf2 and its response to H_2_S treatment in a cellular model of acute infection with *Mycoplasma fermentans*.

**Fig. 1:**
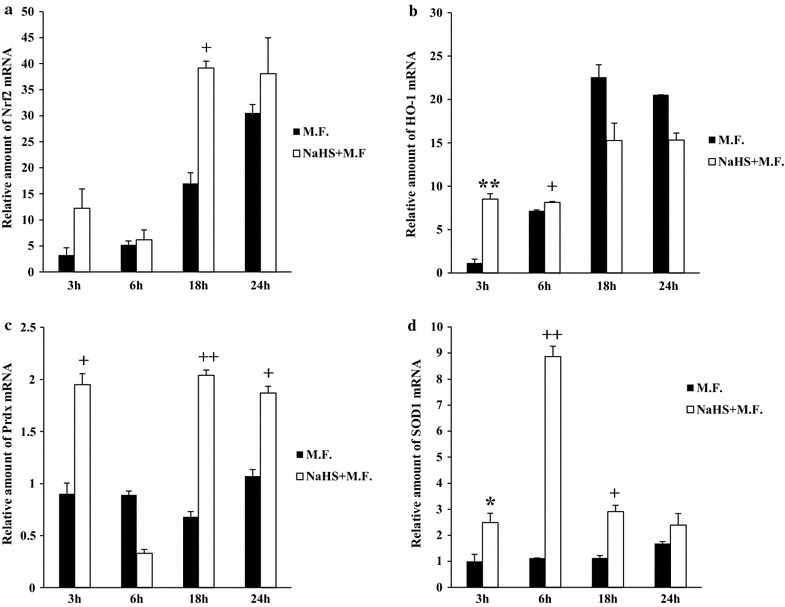
Induction of Nrf2 (**a**), HO-1 (**b**), Prdx (**c**) and SOD1 (**d**) mRNA by H_2_S in *Mycoplasma fermentans* infected U937 cells, analyzed by real time RT-PCR. RNA samples from Mycoplasma-infected U937 cells (M.F.) were collected at 3, 6, 18 and 24 h after NaHS treatments. *Bars* denote the standard deviation. The *histograms* shown are representative of data from three different experiments, with samples treated in duplicate, and normalized to endogenous GAPDH mRNA. The p values were calculated as unequal variance t test of Mycoplasma infected cells relative to Mycoplasma infected cells treated with NaHS: ^++^p ≤ 0.001, ^+^p ≤ 0.01, **p ≤ 0.005, *p ≤ 0.05

To test the hypothesis that H_2_S activated the Nrf2 pathway, a human monocytic cell line (U937) was infected with *Mycoplasma fermentans* and concomitantly treated with NaHS, a fast releasing H_2_S donor, usually used for short term treatments (up to 24 h), at non-toxic concentration of 1 mM. Cells were harvested at several time points (3, 6, 18 and 24 h) after the infection/treatment. Uninfected cells were used as negative controls. RT-PCR analysis was used to monitor the expression of Nrf2 at the different time points. We observed an increase in Nrf2 expression in U937 Mycoplasma-infected cells over time, and this effect was enhanced by the treatment with H_2_S donor. No statistically significant difference was observed after 3 and 6 h of infection/treatment, while a statistically significant increase was observed at 18 h (p ≤ 0.01) (Fig. 1a). To substantiate these results, a real time RT-PCR assay was employed to measure the expression of the Nrf2-ARE inducible detoxifying enzymes: HO-1 (heme oxygenase 1), Prdx (peroxiredoxin) and SOD1 (superoxide dismutases 1). We observed significant increases of all three enzymes mRNA expression levels, though at different time points. The mRNA level of HO-1 increased noticeably after 3 h of infection following NaHS treatment (8.5 versus 1.15 fold in treated versus not-treated, respectively) (p ≤ 0.005) while the effect of H_2_S on the mRNA level of HO-1 was less noticeable at late time points (18 and 24 h) (Fig. 1b). We also observed increased SOD1 mRNA levels in Mycoplasma infected cells treated with NaHS. The highest increase was observed after 6 h with 8.86-fold peak, versus 1.13-fold increase in the corresponding Mycoplasma-infected cells (p ≤ 0.001) (Fig. 1d). In contrast, Prdx mRNA levels were significantly increased at 18 h (2.04 fold increase versus 0.68-fold increase in the not treated cells) (p ≤ 0.001) and 24 h (1.87-fold increase versus 1.07-fold increase in the not treated cells) (p ≤ 0.01). Consequently, Prdx might play a protective role at these late time points. Taken together, these data indicate that the Nrf2 pathway is stimulated by exogenous H_2_S in Mycoplasma-infected cells, leading to increased production of antioxidant/detoxificant enzymes.

To determine whether H_2_S treatment, and the consequent increased production of antioxidant/detoxificant enzymes, had an effect on levels of ROS (Reactive Oxygen Species), well known cell-damaging oxidative agents, U937 cells were infected with Mycoplasma and simultaneously treated with 0.1 mM GYY4137, a water-soluble molecule that, unlike NaHS, decomposes slowly to generate small amounts of H_2_S both in vitro and in vivo [90]. This H_2_S donor allowed us to perform long term experiments for a period up to 6 days.

**Fig. 2:**
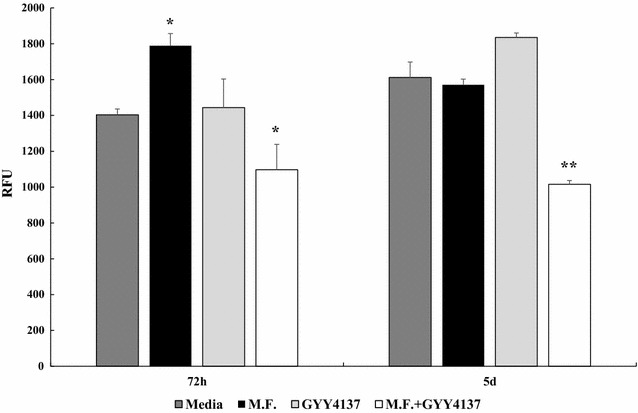
Intracellular ROS assay in U937 cells simultaneously infected with Mycoplasma (M.F.) and treated with GYY4137 at two different time points (72 h and 5 days). The DCF fluorescence intensity is proportional to the ROS levels within the cell cytosol. *Bars* denote the standard deviation. The *histograms* shown are representative of data from three different experiments, with samples analyzed in duplicate. The p values were calculated as unequal variance t test of Mycoplasma infected cells relative to non-infected cells used as control (*p ≤ 0.05) and as unequal variance t test of Mycoplasma infected cells relative to Mycoplasma infected cells treated with GYY4137 (**p = 0.002, *p ≤ 0.05)

Reactive oxygen species levels were measured at different time points (24, 48, 72 h, 5 and 6 days) using a marker for oxidative stress (DCFH-DA). In early time points (24 and 48 h) and in late time points (6 days) there was no significant change in ROS levels in all the samples analyzed (data not shown). In contrast, upon Mycoplasma infection we measured a significant increase of ROS production after 72 h, compared to non-infected cells (p ≤ 0.05) (Fig. 2). At the same time point, there was a significant decrease in ROS production in the cells treated with GYY4137 (p ≤ 0.05) (Fig. 2). Cells treated with GYY4137 and infected with Mycoplasma showed a very statistically significant reduction of ROS production after 5 days (p = 0.002) (Fig. 2).

Our data demonstrate the positive effect on antioxidant/detoxifying cellular functions exerted by H_2_S treatment during acute bacterial infection. Previous studies demonstrated a protective role of H_2_S on various cell types against oxidative stress [91], though the mechanism(s) are not clearly understood. A possible hypothesis is that H_2_S acts both as a direct scavenger of ROS and as an inducer of endogenous antioxidant defenses. The date presented here indicate that in infected monocytes, at least partially, H_2_S antioxidant effects are related to Nrf2/HO-1 pathway upregulation.

While other groups have shown the ability of H_2_S to upregulate cellular antioxidants in an Nrf2-dependent manner in other tissues [92, 93], this is the first time that the same effect has been shown in monocytes/macrophages infected by a bacteria. It is worth noting that macrophages are not only key players in the initiation of inflammation during microbial infection, but also orchestrate its resolution.

**Fig. 3:**
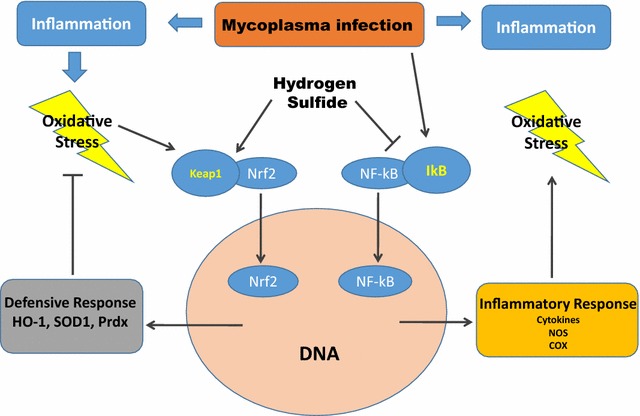
Effects of H_2_S on the Nrf2-dependent antioxidant response and NF-κB pathways, activated by Mycoplasma infection

Our previous studies have shown that H_2_S has an anti-inflammatory effect in Mycoplasma infected monocytes through inhibiting NF-κB pathway and the release of pro-inflammatory cytokines. Furthermore, we have shown that H_2_S improves redox homeostasis in the same model via the activation of Nrf2 pathway. Considering the functional cross-talk between these two important pathways, we propose the possible effectiveness of H_2_S in helping immune cells to regulate the fine balance of cellular redox status and responses to stress and inflammation due to bacterial infection (Fig. 3).

An important question that remains unanswered relates to the mechanism by which H_2_S induces Nrf2 activity. It has been proposed that H_2_S induces modification of critical cysteine residues in Keap1, which results in the release of Nrf2 [94]. Whether H_2_S alters Keap1 and/or Nrf2 directly or through upstream signaling, and how this can modulate NF-κB activity, will require further studies.

## Conclusions

Our data show that H_2_S inhibits the activation and the nuclear translocation of the NF-κB, reducing the transcription of pro-inflammatory genes. Moreover, it enhances Nrf2 functions by activating downstream enzymes such as HO-1 and SOD1, and by decreasing intracellular ROS levels.

Taken together, these results highlight the protective role of H_2_S as anti-inflammatory agent and support the ongoing efforts to develop new H_2_S-releasing compounds as novel therapeutic agents to reduce inflammation. Such compounds could be used for the treatment of conditions where chronic inflammation plays a major role in exacerbating cellular damage in conditions such as arthritis, inflammatory bowel disease, myocardial dysfunction and chemoprevention of cancer [95].

## Methods

### Mycoplasma strains and culture conditions

*Mycoplasma fermentans* PG18 (ATCC, American Type Culture Collection) was grown in 243 media: heart infusion broth (BD) media supplemented with 20% heat inactivated horse serum and 10% yeast extract solution (Invitrogen, Grand Island, NY, USA), at 37 °C, and with 5% CO_2_ in aerobic conditions. Mycoplasma cultures were harvested in late log phase and collected by centrifugation (10 min at 10,000*g* at 4 °C), and washed three times with PBS before using. Cells were infected with Mycoplasma at a concentration of 2 CFU/cell.

U937 cells were grown in RPMI 1640 Medium (Invitrogen, Grand Island, NY, USA) supplemented with 10% fetal bovine serum (FBS) (Gemini Bio-Products, Burlington, Ontario, Canada).

### H_2_S treatments

Cells were treated with two different H_2_S donors: NaHS (Sigma-Aldrich, St. Louis, MO, USA) at 1 mM and GYY4137 (Santa Cruz Biotechnology, Dallas, TX, USA) at 100 μM final concentrations, respectively. NaHCO_3_ (Sigma-Aldrich, St. Louis, MO, USA) and PBS (Invitrogen, Grand Island, NY, USA) were used as controls for NaHS and GYY4137, respectively. Reagents were added directly into the culture medium at optimal and non-toxic concentrations at the time of Mycoplasma infection. NaHS and GYY4137 concentrations were determined by treating U937 with 0.02, 0.1, 0.5, 1 or 2 mM NaHS and GYY4137 for 24 h. NaHS and GYY4137 range of concentrations were not toxic in our experimental conditions and comparable to the physiological level of H_2_S in the organism [96]. Cell viability was determined by flow cytometry analysis after propidium iodide (PI) (2.5 μg/ml) staining (cell exclusion).

All data have been normalized using not infected and not treated cells as control.

### Real time quantitative RT-PCR (qRT-PCR)

For real time PCR assay, U937 cells (1 × 10^6^ cells/well in 6 wells-plates) were infected with *Mycoplasma fermentans*, treated with the optimal concentrations of sulfide donors and then collected at 3, 6, 18 and 24 h after the infection/treatments. RNA was extracted with the RNeasy Mini Kit (Qiagen, Frederick, MD, USA), an aliquot (2 μg) was reverse transcribed (iScript cDNA Synthesis Kit, BioRad, Hercules, CA, USA) and then subjected to real time RT-PCR using the iQ SYBR Green Supermix (BioRad, Hercules, CA, USA).

The cDNAs were amplified with specific primers: GAPDH (Glyceraldehyde 3-phosphate dehydrogenase) (used as housekeeping gene control for Ct determination): forward 5′-CCATGGAGAAGGCTGGGG-3′, reverse 5′-CAAAGTTGTCATGGATGACC-3′; Nrf2: forward 5′-ACACGGTCCACAGCTCAT-3′, reverse 5′-CAGCTCATACTCTTTCCGTCG-3′; HO-1: forward 5′-ATGCCCCAGGATTTGTCAGA-3′, reverse 5′-GAAGACTGGGCTCTCCTTGT-3′; Prdx: forward 5′-AAAGCCACAGCTGTTATGCC-3′, reverse 5′-AAGCACCAATCACTTGGCAG-3′; SOD1: forward 5′-CTAGCGAGTTATGGCGACGA-3′, reverse 5′-CCACACCTTCACTGGTCCAT-3′. Primers were selected by using the NCBI/primer-blast program (www.ncbi.nlm.nih.gov/tools/primer-blast/), synthesized and desalted-purified (Sigma-Aldrich, St. Louis, MO, USA). Amplification (30 s of denaturation at 95 °C, 35 s of annealing at 65 °C and 30 s of extension at 72 °C) was performed for 35 cycles with specific primers for SOD1. The same protocol was used with specific primers for Nrf2, HO-1 and Prdx, except for the annealing temperature that was set at 67 °C. PCR amplification with the primers specific for GAPDH was performed using the following protocol: 28 cycles of 30 s at 94 °C, 35 s at 60 °C and 30 s at 72 °C. All reactions were run in duplicate. Semi-quantitative analysis was based on the cycle number (Ct) whereby the SYBR Green fluorescent signal crossed a threshold in the log-linear range of the RT-PCR. The fold change in Nrf2, HO-1, Prdx and SOD1 mRNA in the U937 cell line was compared with the uninfected control cells at time of infection (time zero), and is shown normalized over GAPDH mRNA measured as internal control.

### Intracellular reactive oxygen species (ROS) assay

The accumulation of ROS within the cells coupled with an increase in oxidative stress was measured using the OxiSelect Intracellular ROS Assay Kit (Green fluorescence) (Cell Biolabs, Inc, San Diego, CA, USA). The assay employs the cell-permeable fluorogenic probe 2′,7′-dichlorodihydrofluorescin diacetate (DCFH-DA). DCFH-DA is diffused into cells and is deacetylated by cellular esterases to non-fluorescent 2′,7′-dichlorodihydrofluorescin (DCFH), which is rapidly oxidized to highly fluorescent 2′,7′-dichlorodihydrofluorescein (DCF) by ROS. The fluorescence intensity is proportional to the ROS levels within the cell cytosol. The effect of antioxidant or free radical compounds on DCF-DA was measured against the fluorescence of the provided DCF standard. U937 cells were plated in duplicate wells at a concentration of 25 × 10^4^ cells/well and were harvested at different time points after *Mycoplasma fermentans* infection and H_2_S treatment: 24, 48, 72 h, 5 and 6 days. At each time point, the cells were washed and incubated for 1 h at 37 °C with 100 µl of DCFH-DA, then washed and lysed for 5 min using reagents provided in the kit. The fluorescence was read at 480 nm excitation/530 nm emission and compared to that of the DCF standard curve.

## Abbreviations

H_2_S: hydrogen sulfide
NaHS: sodium hydrosulfide
GYY4137: morpholin-4-ium-4-methoxyphenyl-(morpholino)-phosphinodithioate
NF-kB: nuclear factor-kB
M.F.: *Mycoplasma fermentans*
Nrf2: nuclear factor (erythroid-derived 2)-like 2
HO-1: heme oxygenase 1
Prdx: peroxiredoxin
SOD1: superoxide dismutases 1
ROS: reactive oxygen species
